# *Pneumocystis jirovecii* Pneumonia in Tropical and Low and Middle Income Countries: A Systematic Review and Meta-Regression

**DOI:** 10.1371/journal.pone.0069969

**Published:** 2013-08-02

**Authors:** David M. Lowe, Molebogeng X. Rangaka, Fabiana Gordon, Chris D. James, Robert F. Miller

**Affiliations:** 1 Wellcome Trust Centre for Research in Clinical Tropical Medicine, Department of Medicine, Imperial College London, London, United Kingdom; 2 Clinical Infectious Disease Research Initiative, Institute of Infectious Diseases and Molecular Medicine, University of Cape Town, Cape Town, South Africa; 3 Statistical Advisory Service, Imperial College London, London, United Kingdom; 4 World Health Organisation Western Pacific Regional Office, Manila, Philippines; 5 Clinical Research Department, Faculty of Infectious and Tropical Diseases, London School of Hygiene and Tropical Medicine, London, United Kingdom; 6 Research Department of Infection and Population Health, Institute of Epidemiology and Healthcare, University College London, London, United Kingdom; Vanderbilt University, United States of America

## Abstract

**Objective:**

*Pneumocystis jirovecii* pneumonia (PCP), the commonest opportunistic infection in HIV-infected patients in the developed world, is less commonly described in tropical and low and middle income countries (LMIC). We sought to investigate predictors of PCP in these settings.

**Design:**

Systematic review and meta-regression.

**Methods:**

Meta-regression of predictors of PCP diagnosis (33 studies). Qualitative and quantitative assessment of recorded CD4 counts, receipt of prophylaxis and antiretrovirals, sensitivity and specificity of clinical signs and symptoms for PCP, co-infection with other pathogens, and case fatality (117 studies).

**Results:**

The most significant predictor of PCP was *per capita* Gross Domestic Product, which showed strong linear association with odds of PCP diagnosis (p<0.0001). This was not explained by study design or diagnostic quality. Geographical area, population age, study setting and year of study also contributed to risk of PCP. Co-infection was common (444 episodes/1425 PCP cases), frequently with virulent organisms. The predictive value of symptoms, signs or simple tests in LMIC settings for diagnosis of PCP was poor. Case fatality was >30%; treatment was largely appropriate. Prophylaxis appeared to reduce the risk for development of PCP, however 24% of children with PCP were receiving prophylaxis. CD4 counts at presentation with PCP were usually <200×10^3/^ml.

**Conclusions:**

There is a positive relationship between GDP and risk of PCP diagnosis. Although failure to diagnose infection in poorer countries may contribute to this, we also hypothesise that poverty exposes at-risk patients to a wide range of infections and that the relatively non-pathogenic *P. jirovecii* is therefore under-represented. As LMIC develop economically they eliminate the conditions underlying transmission of virulent infection: *P. jirovecii*, ubiquitous in all settings, then becomes a greater relative threat.

## Introduction

*Pneumocystis jirovecii* pneumonia (PCP) is one of the commonest opportunistic infection in Human Immunodeficiency Virus (HIV)-infected patients in the developed world (historically the leading AIDS-indicator disease in both the United States and Europe) [1], but has always been regarded as rare in low and middle income countries (LMIC) predominantly of tropical latitude [2], [3]. However, the organism is ubiquitous and seroprevalence data indicates worldwide exposure [4]–[7]. This suggests that epidemiological differences are not explained by geography, nor are they easily attributable to genetic differences in the organism or the host [8], [9]. Factors influencing prevalence in these settings have not been systematically evaluated.

Our primary objective was to establish major determinants of PCP in HIV-infected individuals in tropical and LMIC. The ‘gold’ diagnostic standard of cytological examination of respiratory samples may be less available in LMIC, affecting reported prevalence. Gross domestic product (GDP), a measure of population income level, influences prevalence of HIV and opportunistic infections including tuberculosis [10], [11], and an association with PCP may thus be discovered. Relationships with time, geographical area or study setting may reflect genuine epidemiology [12], but might also reflect varying awareness of the condition among clinicians [13].

Additionally, assessment of the accuracy of symptoms, signs, or laboratory tests in LMIC for diagnosis of PCP, rates of co-infection that may confound diagnosis [14], PCP case fatality, CD4 count at presentation, treatment strategies, and efficacy of prophylaxis or antiretroviral therapy (ART) in reducing risk of PCP was undertaken.

## Methods

### Search Strategy and Study Selection

Table 1 details the search strategy. Summaries of AIDS-defining illnesses (ADI) or hospitalisations in the HIV-infected population were included for primary analysis, since these were considered most representative of overall disease burden and their broad entry criteria carried minimal risk of bias. Presumptive diagnoses were included, consistent with practice in many centres [1], although quality of diagnosis was also assessed. All HIV-infected participants in the studies were included. DML extracted and RFM verified the data.

**Table 1:** Search strategy.

**Databases searched**	PubMed database (provided by the US National Library of Medicine)
	Cochrane Library
	African Journals Online
**First/Last performed**	March 2006/January 2011
**Search terms**	pneumocystis, carinii, jiroveci, jirovecii, PCP, PJP
**Cross-referenced with:**	Africa, Asia, Middle East, India, South America, Central America, Latin America, developing world, tropics
**Study designs included**	Prospective or cross-sectional clinical studies
	Retrospective reviews of medical records/databases
	Cohort studies
	Autopsy/mortality studies
**Data extracted**	Year(s) studied
	Year published
	Study methodology
	Country
	Specific study setting and population
	Method(s) of diagnosis for PCP
	Total number of patients included
	Total number of patients with HIV
	Number of patients with PCP (sub-divided according to HIV status)
	Total number of patients with tuberculosis (TB) (sub-divided according to HIV status)
	Specific clinical findings in patients with PCP
	Prior receipt of antiretrovirals amongst patients with and without PCP
	Prior receipt of PCP prophylaxis amongst patients with and without PCP
	CD4 count in patients with PCP
	Co-infection in patients with PCP
	Mortality amongst patients with PCP
	Treatment strategy of patients with PCP
**Exclusion criteria**	Published before 1985
	Non-English language
	Study setting outside pre-specified geographical areas (Central or South America, East or South-East Asia, Indian sub-continent, Middle East, Africa)
	Definite subset of data published elsewhere (possible overlap of patients is indicated in relevant tables)
	Unable to extract data

PCP/PJP = *Pneumocystis jirovecii* pneumonia; HIV = Human Immunodeficiency Virus; CD4 = Cluster of Differentiation 4.

### Quality Assessment

A modified Newcastle Ottawa Scale (NOS) was used to assess selection and comparability of patients (Table 2). Since most studies in the primary analysis did not control for specific features, comparability was assessed on the basis of identical recruitment of PCP and non-PCP cases. Quality of outcome ascertainment was assessed by a quantitative diagnostic scoring system (Table 2). Clinical diagnosis only scored ‘1’; diagnosis based solely on spontaneously expectorated sputum scored ‘2’. Values between 3 and 5 indicate that appropriate advanced diagnostics were available and used for some, but not all, patients. A score of ‘6’ was awarded if all patients were diagnosed with high accuracy. Polymerase Chain Reaction (PCR) methods for diagnosing PCP were only being validated towards the end of the time range of these studies. Correspondingly, accuracy was not established and no studies used PCR alone without either immunofluorescence or cytology for diagnosis: for these reasons, PCR is not included in the scoring system.

**Table 2:** Quality scores for studies included in primary analysis.

Study	Selection score (1 point per item)	Comparability score (1 point per item)	Diagnostic score	
	- Inclusion/Exclusion criteria clearly stated?	- PCP and non-PCP cases recruited identically?	**Samples/diagnostic tests**	**Score**
	- Cases clearly representative (eg consecutive)?	- Patients on/not on prophylaxis recruited identically? (*Where applicable*)	Clinical diagnosis (empiricallydiagnosed)	**1**
	- Numbers/reasons for non-inclusion clearly stated?		Clinical diagnosis (empiricallydiagnosed) or histochemical/IFstaining of spontaneouslyexpectorated sputum/NPA	**2**
	- Method for ascertaining whether PCP prophylaxis was used clearly stated and acceptable? (*Where applicable*)		Clinical diagnosis (empiricallydiagnosed) or histochemical/IFstaining of induced sputum	**3**
			Clinical diagnosis (empiricallydiagnosed) or histochemicalstaining of BAL fluid	**4**
			Clinical diagnosis (empiricallydiagnosed) or histochemical/IFstaining of induced sputum orhistochemical staining of BALfluid or lung tissue (TBB/OLB)	**5**
			Histochemical staining/IF ofinduced sputum or histochemicalstaining of BAL fluid or lungtissue (TBB/OLB) – all patients	**6**
**Moreira-Junior ** [**52**]	3	1	5	
**Udwadia ** [**53**]	2	1	6	
**Sharma ** [**54**]	3	1	5	
**Bellamy ** [**55**]	3	1	NR	
**Nissapatorn 2003 ** [**56**]	1	1	1	
**Singh ** [**57**]	0	1	2	
**Kim ** [**58**]	0	1	NR	
**Senya ** [**20**]	2	1	1	
**Inverarity ** [**59**]	2	1	1	
**Kumarasamy ** [**60**]	1	1	NR	
**Louie ** [**61**]	2	1	6	
**Anekthananon ** [**37**]	2	1	NR	
**Fang ** [**62**]	3	1	5	
**Oh ** [**63**]	2	1	5	
**Tansuphasawadikul ** [**64**]	3	1	1	
**Yoong ** [**41**]	1	1	NR	
**George ** [**65**]	2	1	1	
**Siegman-Igra ** [**44**]	3	1	NR	
**Nissapatorn 2004 ** [**66**]	1	1	1	
**Lian ** [**22**]	3	1	NR	
**Santos ** [**67**]	2	1	NR	
**al-Haddad ** [**45**]	2	1	NR	
**Tan ** [**68**]	3	1	NR	
**Swasdisevi ** [**69**]	0	1	1	
**Vithayasi ** [**70**]	0	1	NR	
**Kong ** [**71**]	2	1	5	
**Chariyalertsak ** [**25**]	1	1	1	
**Wong ** [**72**]	2	1	NR	
**Shah ** [**73**]	2	1	NR	
**Rajasekaran ** [**74**]	2	1	1	
**Solomon ** [**75**]	2	1	NR	
**Soares ** [**76**]	3	1	1	
**Bedri ** [**77**]	3	1	1	

PCP – *Pneumocystis jirovecii* pneumonia; IF = Immunofluorescence; NPA = nasopharyngeal aspirate; BAL = bronchoalveolar lavage; TBB = trans-bronchial biopsy; OLB = open lung biopsy; NR = Not Recorded.

### Bias and Confounding

Within-study bias mostly derived from entry criteria specifying pre-existing diseases or clinical characteristics. Studies with biased entry criteria were excluded from pooled analysis. Increased clinical diagnosis of PCP resulting from greater awareness among clinicians may have created bias, but we could not formally assess this. We planned to control for time and geographical area in an attempt to eliminate or identify this bias. Population characteristics may vary depending on the type of research institution and so we also aimed to control for this variable.

### Analysis and Statistics

Primary analysis was performed using logistic regression. The dependent variable was diagnosis of PCP (present/absent). Categorical predictor variables were study population (adult, paediatric, combined, unspecified); geographical area (Africa, Middle East, Indian sub-continent, East/South-East Asia, Central/South America) and study setting (rural/primary care, hospital, tertiary/specialist/University hospital, nationwide). Quality assessments (selection score and diagnostic score) were treated as categorical predictors. Due to low numbers within some diagnostic score groups, values were combined into categories of ‘Low quality’ (score 1 or 2), ‘High quality’ (score 5 or 6) or ‘Not Recorded’. *Per capita* GDP in ‘constant US dollars’ (after controlling for inflation) was obtained from the World Bank’s world development indicators for the median year of the study [15]. Median year of study and GDP were treated as continuous covariates. Year was centred to its mean; GDP showed a skewed distribution and was centred to its median. When the effects of GDP and year were seen to be non-linear a quadratic term was added. Relative importance of model components was assessed via Chi-square improvement (based on -2×Log Likelihood) with iterative addition of predictors.

Case fatality was assessed using logistic regression with death/survival as the dependent variable and the same predictors (as above). Odds Ratios for the effect of prophylaxis were calculated using Mantel-Haenszel methodology. A forest plot was used to assess heterogeneity in prevalence in studies selected for primary analysis; heterogeneity in case fatality and prophylaxis data was assessed via I^2^ analysis. Statistics were performed using SPSS version 18.0; meta-analysis functions were performed with Review Manager Version 5.1 (Cochrane Collaboration 2011, Copenhagen).

## Results

**Figure 1:**
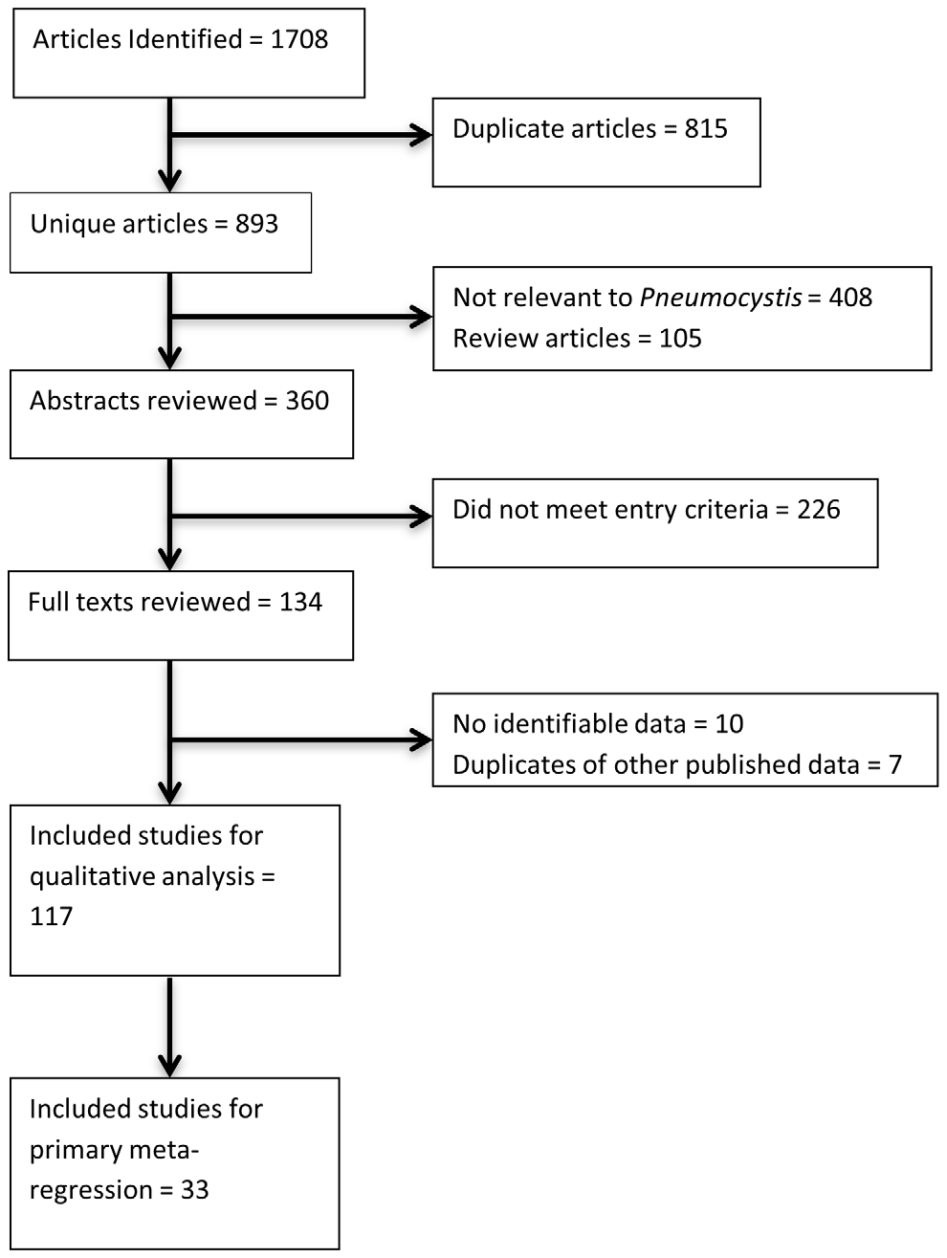
Results of literature search.

Of 1708 citations identified, 134 full-text papers were reviewed. Ten contained no identifiable data and seven were subsets of data published elsewhere (see Figure 1 for a PRISMA [16] flowchart; see Checklist S1 for the PRISMA checklist). Table 3 summarises the remaining 117 studies. Papers reviewed but not specifically cited in the text are included as Supplementary References (References S1). Eleven studies were from high income countries and 106 were from LMIC. Seventy-eight studies (67%) were performed in University/tertiary/specialist centres, 25 (21%) were performed in other (or undefined) hospital settings, five (4%) in rural healthcare facilities, seven (6%) were nationwide/regional studies and two (2%) reported occupational health services at mines.

**Table 3:** Classification of reviewed studies.

	Africa		Indian sub-continent		Central and South America		East and South-East Asia		Middle East	
**Respiratory studies**	Adult	18	Adult	2	Adult	1	Adult	7		
	Paed	11			Paed	1	Paed	1		
	Unspec	1								
**Autopsy studies**	Adult	3	Adult	1	Adult	3	Paed	1		
	Paed	6	Paed	1	Comb	2				
**Other mortality data**	Adult	4			Adult	1	Paed	1		
**AIDS defining illnesses or**	Paed	1	Adult	6	Adult	3	Adult	17	Adult	2
**reasons for**			Paed	2	Comb	1	Comb	2		
**hospitalisation in HIV infected population**			Comb	1						
**HIV Cohort studies**	Adult	1	Paed	6	Adult	1	Adult	3	Adult	1
	Paed	1	Comb	1	Paed	1	Comb	2		
**Non-HIV immunocompromised populations** 1							Adult	3		

1 Not included in analysis.

AIDS = Acquired Immunodeficiency Syndrome; HIV = Human Immunodeficiency Virus; Paed = paediatric; Comb = combined adult and paediatric; unspec = unspecified age group.

**Figure 2:**
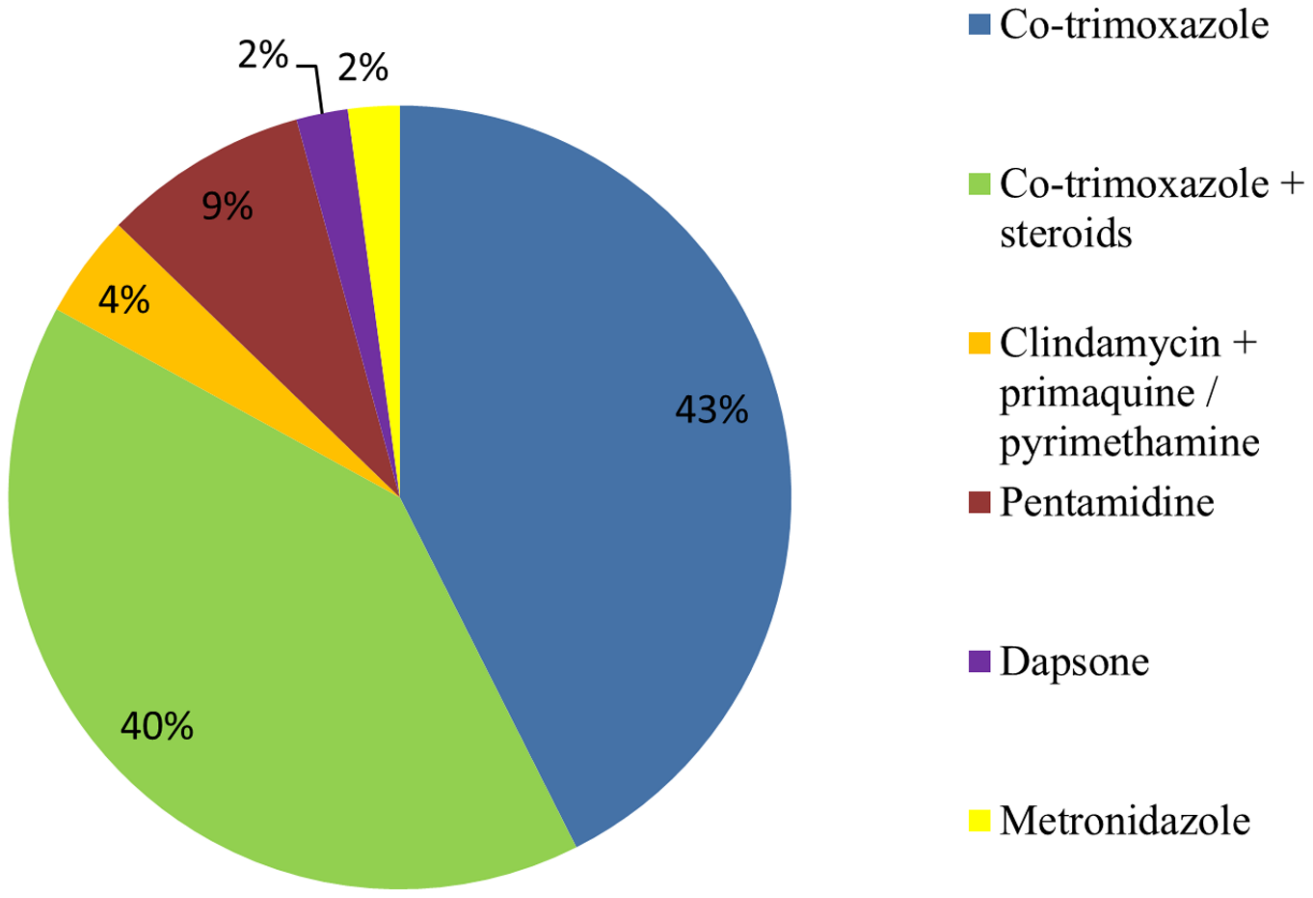
Treatment strategies for PCP in reviewed studies. Total strategies for this analysis = 46.

Forty-six studies declared their treatment for PCP (Figure 2): 39 used co-trimoxazole, of which 19 mentioned concomitant use of steroids; four used pentamidine, two used clindamycin plus primaquine or pyrimethamine and one used dapsone. There were few data on the effect of ART on PCP prevalence. Several papers documented that no patients were taking ART at the time of presentation [17]–[21]. Lian *et al* [22] demonstrated a decline in PCP incidence from 21/128 (16.4%) prior to availability of ART to 1/128 (0.78%) subsequently; Kouakoussui *et al* [23] reported two cases of PCP in their cohort before availability of ART and none after. However, Brazilian mortality data [24], reported that 81 of 403 (est.) deceased PCP patients had received ART.

### PCP Prevalence is more Dependent on *per capita* Gross Domestic Product than any Other Factor

**Figure 3:**
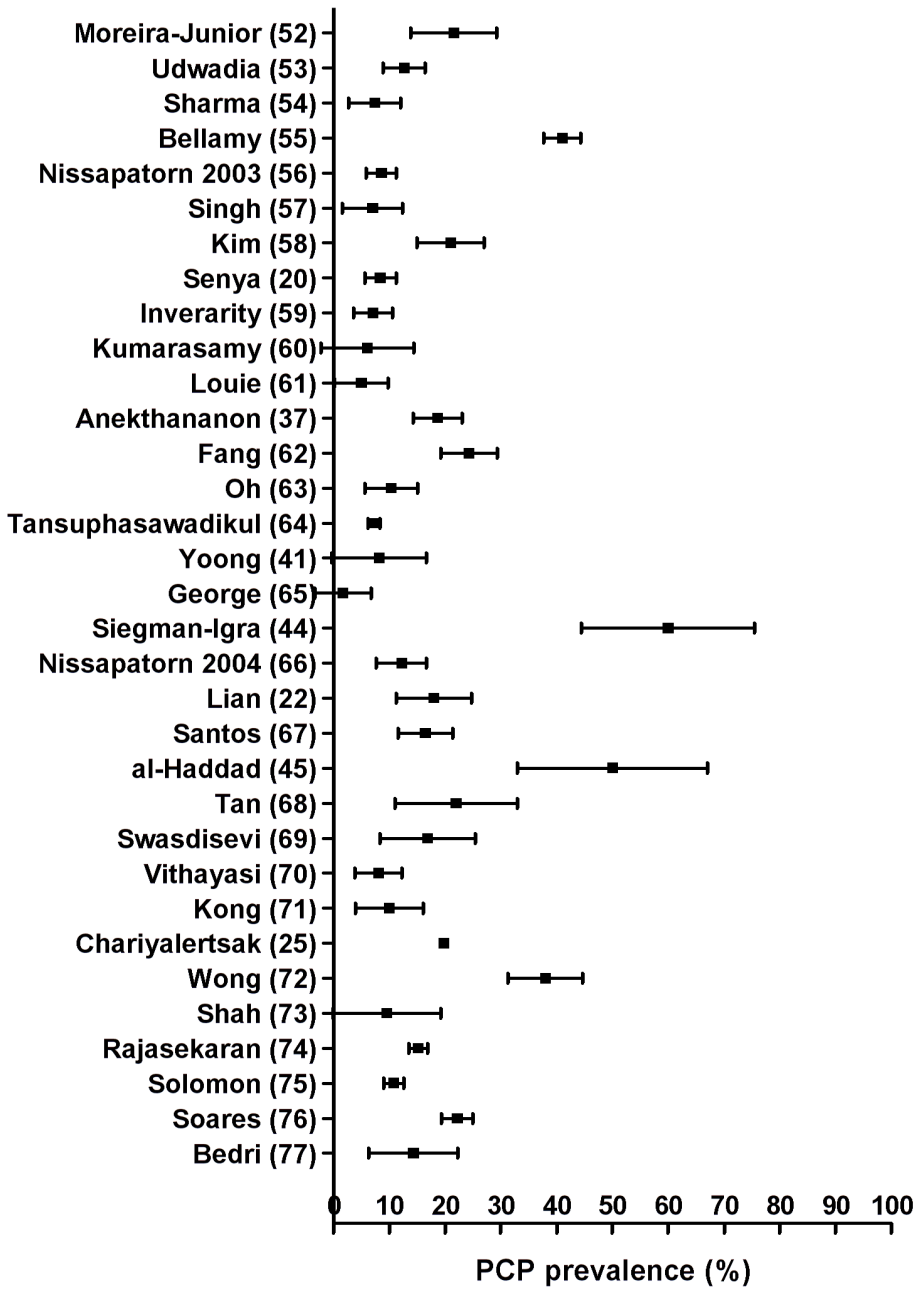
Prevalence of PCP in studies included for primary analysis, indicating heterogeneity of data set. Confidence intervals for point prevalences were determined by adjusted Wald method.

Thirty-five studies detailed ADI or reasons for hospitalisation within an HIV-infected population, but two pre-specified ‘fever of unknown origin’ and were excluded from primary analysis due to recruitment bias. The remaining 33 studies represented 114,389 people and 21,853 PCP diagnoses. However, one Thai surveillance study [25] accounted for 101,945 people and 20,145 diagnoses. Results are reported including this study, since removing it from analysis did not significantly alter findings. The overall median year of study was 1996 and median GDP was $2141 *per capita*. The prevalence of PCP as an ADI/reason for hospitalisation ranged from 1.67% to 60.0%. Initial assessment of heterogeneity (Figure 3) suggested that summary measures were inappropriate and we proceeded to meta-regression.

There was a positive linear relationship between median year of study and odds of PCP (adjusted odds ratio (aOR) per year = 1.05, 95% CI 1.03–1.07, p<0.001). Geographical area was a significant predictor, with higher odds for a diagnosis of PCP in Central/South America (aOR = 1.72, 95% CI 1.35–2.19, p<0.001) and the Middle East (aOR = 2.53, 95% CI 1.46–4.39, p = 0.001) versus East/South-East Asia. However, there were only two studies from the Middle East (total n = 63), and so we interpreted this result cautiously. Adult or combined-age studies yielded lower odds for PCP than paediatric studies (for adult studies aOR = 0.67, 95% CI 0.52–0.86, p = 0.002; for combined studies aOR = 0.70, 95% CI 0.56–0.87, p = 0.001). Nationwide surveillance demonstrated higher odds than hospital studies (secondary or tertiary care), with the lowest odds in rural/primary care centres, although only one rural study (n = 225) was included (versus nationwide studies: aOR for tertiary/University hospitals = 0.46, 95% CI 0.35–0.60, p<0.001; aOR for other hospitals = 0.47, 95% CI 0.33–0.68, p<0.001; aOR for rural/primary care studies = 0.28, 95% CI 0.16–0.50, p<0.001).

**Figure 4:**
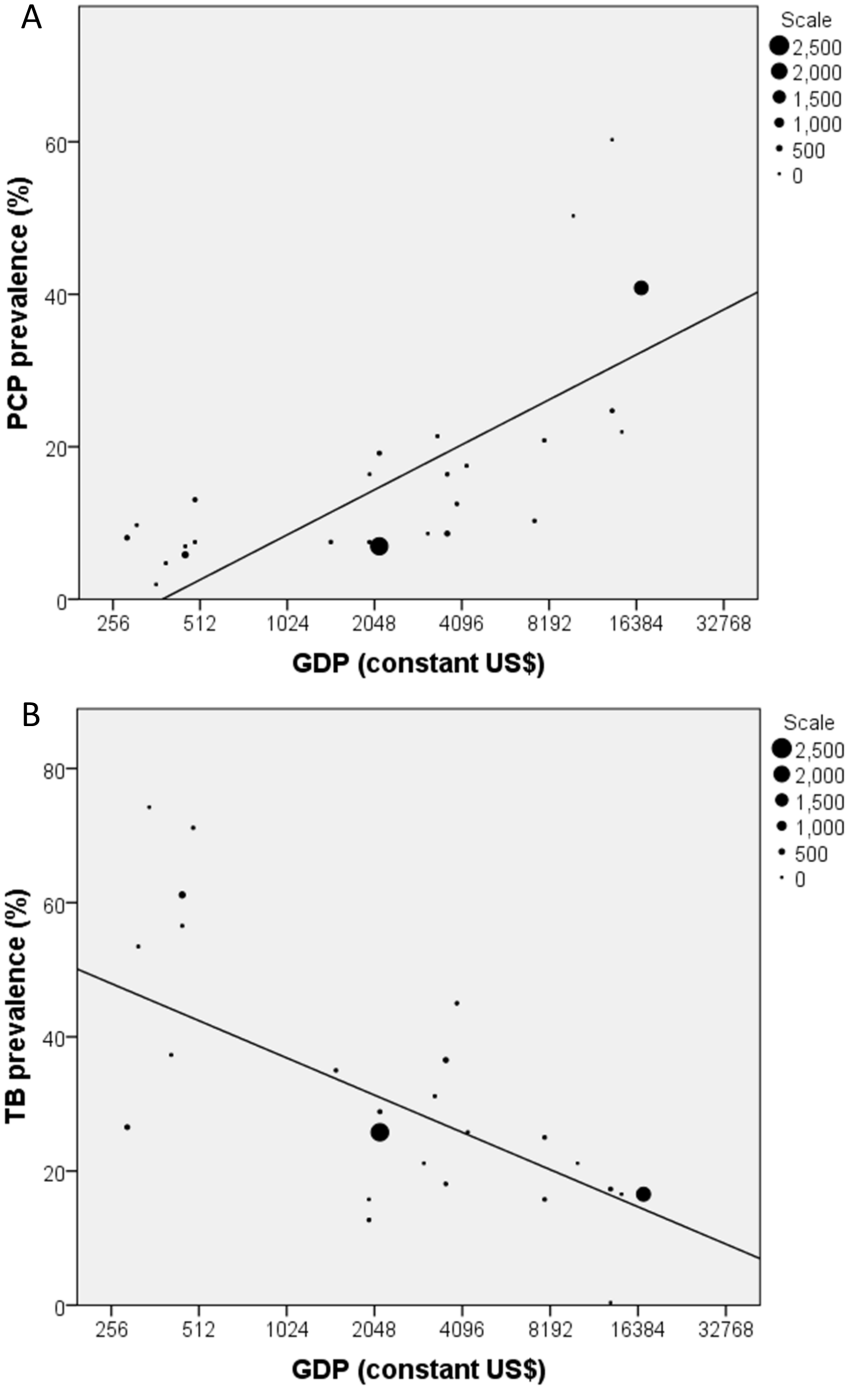
Relationship between prevalence of diagnoses and GDP. A. Relationship between *Pneumocystis jirovecii* pneumonia (PCP) prevalence (%) and *per capita* Gross Domestic Product (constant US$) in adult AIDS-defining illness studies or reasons for hospitalisation in an HIV infected population; size of marker indicates size of study; a regression line is added for clarity. Note the logarithmic x-axis. B. Relationship between tuberculosis (TB) prevalence (%) and *per capita* Gross Domestic Product (constant US$) for the same studies.

GDP was a highly significant linear predictor of a diagnosis of PCP (p<0.0001). An increase of $20,000 (the approximate range of GDPs included) increases the odds for a diagnosis of PCP 10.54-fold (95% CI 8.41–13.20). Chi-square improvement analysis revealed GDP to be the most important predictor of a diagnosis of PCP (order of importance: GDP>population age>geographical area>study setting>median year). Results from logistic regression are summarised in Table 4. Figure 4A provides a graphical representation of prevalence data compared with GDP for adult studies; tuberculosis (TB) prevalence in the same studies is depicted in Figure 4B and shows a decline with increasing GDP, as previously described [11]. In these data, the correlation between PCP prevalence and GDP was 0.78 (p<0.001) unweighted, or 0.94 (p<0.001) weighted by study size; the correlation between TB prevalence and GDP was -0.61 (p = 0.001) unweighted, or -0.57 (p<0.001) weighted by study size.

**Table 4:** Results of primary analysis of predictors of PCP via logistic regression.

		Excluding diagnostic score			Including diagnostic score			
Predictor		Exp(B)	95% CI	p-value		Exp(B)	95% CI	p-value
**Median year (per year)**		1.05	1.03–1.07	<0.001		N/A		
**Median year (quadratic)**		N/A				…^1^		0.002
**GDP (per $1000)**		1.12	1.11–1.14	<0.0001		1.10	1.09–1.12	<0.0001
**Study Population**	**Adult**	0.67	0.52–0.86	0.002		0.35	0.24–0.52	<0.001
	**Combined**	0.70	0.56–0.87	0.001		0.36	0.25–0.50	<0.001
	**Paediatric**	1				1		
**Geographical area**	**Africa**	1.34	0.64–2.81	0.443		0.70	0.32–1.57	0.389
	**India**	0.95	0.77–1.17	0.632		0.65	0.50–0.86	0.002
	**Middle East**	2.53	1.46–4.39	0.001		3.47	1.84–6.55	<0.001
	**Central/South America**	1.72	1.35–2.19	<0.001		2.29	1.70–3.09	<0.001
	**East/South-East Asia**	1				1		
**Study setting**	**Rural/primary care**	0.28	0.16–0.50	<0.001		0.27	0.15–0.49	<0.001
	**Hospital** **(secondary level)**	0.47	0.33–0.68	<0.001		0.45	0.31–0.66	<0.001
	**Hospital (tertiary** **or University)**	0.46	0.35–0.60	<0.001		0.40	0.30–0.54	<0.001
	**Nationwide** **surveillance**	1				1		
**Diagnostic score**	**Low quality**	N/A				0.57	0.46–0.70	<0.001
	**High quality**	N/A				0.76	0.62–0.93	0.007
	**Not Recorded**	N/A				1		

1 Odds defined by Exp(0.068–0.007–2×0.007×[Mean of all study median years – Median year of specific study]).

CI = confidence interval; GDP = per capita Gross Domestic Product (constant US$). N/A = Not Applicable.

Since Africa was under-represented in these data we also analysed the most common African study type, adult clinical studies of sub-acute respiratory illness (note there is some heterogeneity in entry criteria). There were 2109 HIV-infected patients and 277 PCP cases from 18 studies. There was a linear relationship between GDP and odds of PCP (p = 0.021); an increase of $20,000 increased the odds for PCP 37.2-fold (95% CI 1.74–794.2). There were lower odds for PCP in rural settings than hospitals (aOR = 0.28, 95% CI 0.08–0.96, p = 0.042) and a quadratic relationship with time showing a fall in odds followed by a rise over the included years (Table 5).

**Table 5:** Results of analysis of predictors of PCP via logistic regression for 18 African adult clinical respiratory studies.

Predictor		Exp(B)	95% CI	p-value
**Median year (per year)**		N/A		
**Median year (quadratic)**		…^1^		<0.001
**GDP (per $1000)**		1.20	1.03–1.40	0.021
**Study setting**	**Rural/primary care**	0.28	0.08–0.96	0.042
	**Hospital (secondary level)**	1		
	**Hospital (tertiary or University)**	0.73	0.45–1.19	0.203

1 Odds defined by Exp(0.021+0.021+2×0.021×[Median year of specific study – Mean of all study median years]).

CI = confidence interval; GDP = per capita Gross Domestic Product (constant US$). N/A = Not Applicable.

### Quality Assessments do not Explain the Associations Observed

Applying our modified NOS to the primary analysis, we concluded that patient comparability was acceptable since all studies recruited PCP and non-PCP cases identically (Table 2). Each study was also assessed according to the first three Selection parameters and awarded a score from 0 to 3. Selection score was not a significant predictor of PCP diagnosis in logistic regression, and a strong relationship with GDP persisted when analysis was restricted to studies which met two or more criteria (24 studies).

Diagnostic score was a significant predictor of PCP diagnosis in logistic regression (p<0.001). Lowest odds were seen in the ‘Low quality’ group, but both ‘Low quality’ and ‘High quality’ studies showed lower odds than the ‘Not Recorded’ group. However, after including diagnostic score as a predictor the only differences in other results were that the relationship with time became quadratic, plateauing between 2000–2001 (p = 0.002), and that lower odds of PCP were seen in Indian studies compared with East/South-East Asia (Table 4). GDP remained the strongest predictor using Chi-square improvement analysis; an increase in GDP of $1,000 in this model increased the odds for diagnosis of PCP 1.10-fold (95% CI 1.09–1.12). Restricting analysis to studies with high diagnostic quality (7 studies with score 5 or 6) revealed similar results to the overall analysis: an increase in GDP of $1,000 increased the odds for diagnosis of PCP 1.14-fold (95% CI 1.09–1.20, p<0.001). The African clinical studies generally diagnosed PCP with a high degree of certainty, with a Diagnostic Score of ‘6’ for 15 studies, ‘5’ for two studies and ‘2’ for only one study. No correlations existed between quality scores and either GDP or median year, including quality scores as fixed factors and controlling for other predictors in a linear regression model. In particular, there was no significant relationship between GDP and diagnostic score.

### Diagnostic Value of Clinical Symptoms, Signs or Simple Tests are Generally Poor for PCP in Low Income Settings

**Figure 5:**
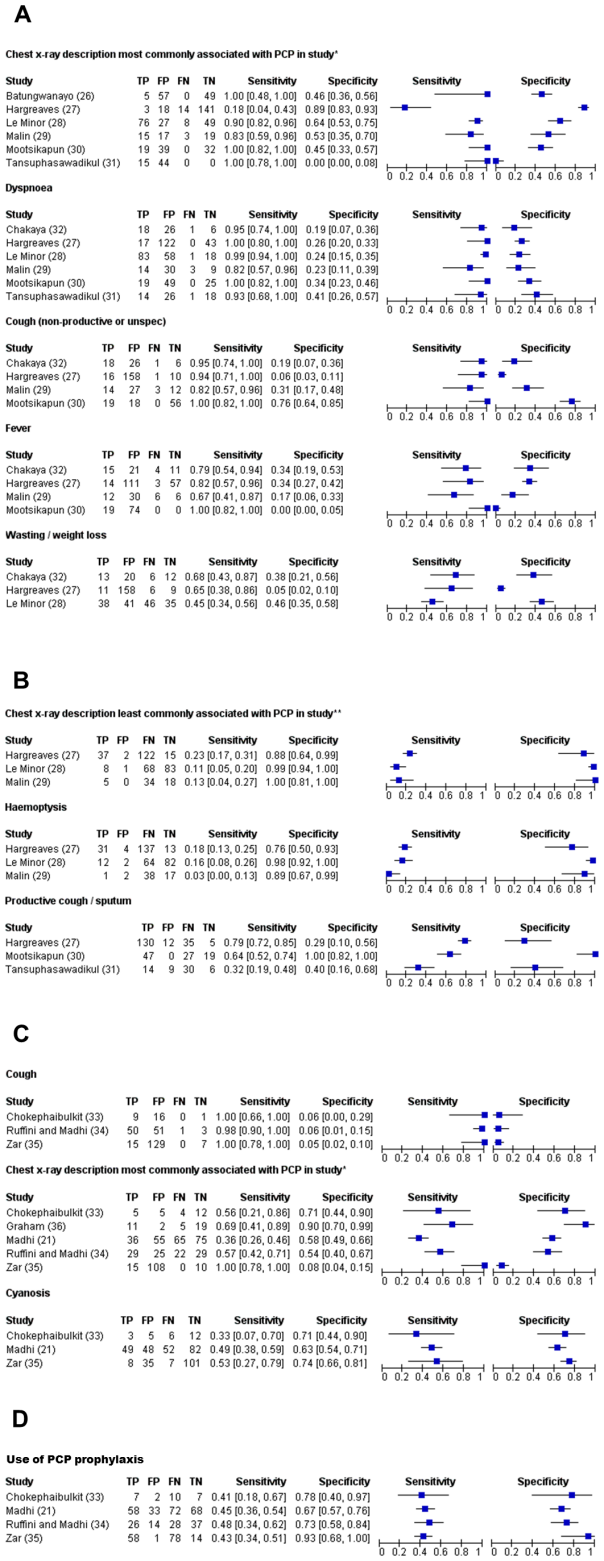
Sensitivity and specificity for *Pneumocystis jirovecii* pneumonia (PCP) of most commonly described signs, symptoms, clinical tests or aspects of history. A. predictive of PCP in adults, B. predictive of absence of PCP in adults, C. predictive of PCP in children, D. predictive of absence of PCP in children. ^*^ Chest x-ray descriptions most commonly associated with PCP in adults: ‘interstitial shadowing’; ‘fine shadowing’; ‘minimally abnormal not TB’; ‘diffuse shadowing’. Chest x-ray descriptions most commonly associated with PCP in children: ‘alveolar consolidation’; ‘interstitial infiltration’; ‘diffuse bilateral alveolar shadowing’; ‘consolidation’. ^**^ Chest x-ray descriptions least commonly associated with PCP in adults: ‘cavities’; ‘hilar lymph nodes’; ‘classical TB’. TP = True Positive; FP = False Positive; FN = False Negative; TN = True Negative.

We evaluated individual symptoms, signs, diagnostic tests and aspects of patient history for their ability to predict the presence or absence of a PCP diagnosis. Seven adult studies [26]–[32] (total 701 patients, 177 with PCP) and five paediatric studies [21], [33]–[36] (total 550 patients, 192 with PCP) were included; parameters detailed in three or more papers are displayed in Figure 5. Pooled estimates are not provided due to high heterogeneity. Indicators of PCP with high sensitivity and negative predictive value, for example cough or dyspnoea, generally had poor specificity and positive predictive value. Certain indicators pointing against a diagnosis of PCP, for example haemoptysis, demonstrated high specificity for a subset without PCP, but poor overall sensitivity for ‘PCP-negatives’.

### Co-infection is Common with PCP in Tropical and LMIC Settings

Table 6 demonstrates documented co-infections among 1,425 PCP patients, derived from 49 studies. These represent infectious rather than patient episodes and frequently a single patient had multiple co-existing infections. Studies whose entry criteria depended on a prior infectious diagnosis other than HIV (eg tuberculosis) were excluded from this analysis. There was a bias towards respiratory pathogens, with many studies basing all analysis on sputum/bronchial washings or including only patients with respiratory symptoms. *M.tuberculosis* accounted for at least 103 of 444 episodes (23.2%). Cytomegalovirus (CMV) was found in 104 episodes (23.4%). Bacterial infection (generally with virulent organisms, where specified) was identified in 116 episodes (26.1%).

**Table 6:** Co-infection in PCP patients.

Pathogen	Number
**Bacteria**	
Bacterial pathogens unspec	30
Bacterial pneumonia unspec	42
Bacterial sepsis/bacteremia unspec	12
*Streptococcus pneumoniae*	6
*Staphylococcus aureus*	10
*Klebsiella* spp	6
*Salmonella* spp	1
*Proteus* spp	1
*Pseudomonas* spp	2
*Haemophilus influenzae*	2
*Corynebacterium* spp	1
*Fusarium* spp	1
*Nocardia* spp	2
**Mycobacteria**	
*Mycobacterium tuberculosis*	103
Myobacteria other than tuberculosis (MOTT)	3
Mycobacteria unspec	6
**Fungi**	
*Cryptococcu*s spp	63
*Aspergillus* spp	4
Invasive candidiasis	7
**Protozoa**	
*Cryptosporidium* spp	1
*Toxoplasma gondii*	1
**Viruses**	
Respiratory virus unspec	26
*Cytomegalovirus*	104
Respiratory syncitial virus	1
*Herpes simplex*	2
*Adenovirus*	2
**Other**	
Infection unspec	5
**Total documented co-infections**	**444**
**Total PCP patients (from these studies)**	**1425**

Unspec = unspecified.

### Case Fatality from PCP in Tropical and LMIC Settings is High, is not Improving with Time, but Negatively Correlates with GDP

Table 7 details case fatality rates among people with a PCP diagnosis in the included studies. Because entry criteria differed between geographical areas, it was not considered valid to compare case fatality rates between regions. There was considerable heterogeneity in these data (I^2^>90% for all datasets except adult mortality in Central/South America), making us interpret pooled estimates cautiously. Nevertheless, as an indicative figure the overall case fatality from all studies in LMIC was 226/733 (30.9%, 95% CI 27.6%–34.3%). Limiting analysis to studies with diagnostic score 5 or 6 (22 studies) revealed a pooled case fatality estimate of 31.4%, and limiting to confirmed diagnoses (Score 6; 16 studies) revealed a pooled estimate of 28.5%. There was no significant time trend in case fatality rates, as assessed by logistic regression (p = 0.894). However, there was a significant negative relationship with GDP, which persisted after controlling for geographical area (the only other significant predictor in univariate analysis, likely confounded by study design). The crude OR for case fatality per $1000 increase in GDP was 0.96 (95% CI 0.92–1.00, p = 0.047), and adjusted OR = 0.86 (95% CI 0.79–0.94, p = 0.001).

**Table 7:** Case fatality amongst people with a diagnosis of PCP in included studies.

Study	Population	Years studied	Case fatality (n dead/n with PCP (%))
**African studies**			
Chakaya *et al* [32]	Adult	1999–2000	5/19 (26.3%)
Malin *et al* [29]	Adult	1992–1993	2/21 (9.52%)
Atzori *et al* [88]	Adult	1991	1/3 (33.3%)
Machiels & Urban [17]	Adult	1990	1/4 (25.0%)
Corbett *et al* [89]	Adult	1998–1999	1/8 (12.5%)
van Oosterhout *et al* [80]	Adult	2002–2004	3/6 (50.0%)
Carme *et al* [90]	Adult	NR	3/5 (60.0%)
Kibiki *et al* [83]	Adult	NR	0/9 (0.0%)
Delport & Brisley [91]	Paediatric	1994–1995	2/2 (100%)
Bakeera-Kitaka *et al* [92]	Paediatric	2001	8/20 (40.0%)
Madhi *et al* [21]	Paediatric	2000–2001	20/100 (20.0%)
Ruffini & Madhi [34]	Paediatric	1999	16/58 (27.6%)
Zar *et al* [39]	Paediatric	1998	8/19 (42.1%)
Graham *et al* [36]	Paediatric	1996	10/16 (62.5%)
Kamiya *et al* [93]	Paediatric	1995	4/5 (80.0%)
Uriyo *et al* [94]	Paediatric	2003	2/2 (100%)
Rabie *et al* [95]	Paediatric	2003	11/18 (61.1%)
Morrow *et al* [85]	Paediatric	2006–2008	17/43 (39.5%)
**South-East/East Asian low and middle income country studies**			
Tansuphasawadikul *et al* [31]	Adult	2002–2003	1/15 (6.67%)
Narata *et al* [96]	Adult	1986–2004	0/2 (0.0%)
Louie *et al* [61]	Adult	2000	1/5 (20.0%)
Swasdisevi [69]	Adult	1994	7/13 (53.8%)
Manaloto *et al* [42]	Adult	1985–1992	5/7 (71.4%)
Ismail *et al* [97]	Combined	1986–1994	3/24 (12.5%)
Chokephaibulkit *et al* [33]	Paediatric	1996–1997	4/9 (44.4%)
**South-East/East Asian high income country studies**			
Lee *et al* [98]	Adult	1985–1996	0/12 (0.0%)
Kim *et al* [58]	Adult	1985–2000	9/37 (24.3%)
Tan *et al* [68]	Adult	1986–1994	1/11 (9.09%)
**Middle East studies**			
Siegman-Igra *et al* [44]	Adult	1982–1987	18/21 (85.7%)
Moses *et al* [87]	Combined	1985–1994	1/14 (7.14%)
**Central/South American studies**			
Weinberg & Duarte [99]	Adult	1988–1999	4/15 (26.7%)
Lambertucci *et al* [100]	Adult	1989–1997	2/6 (33.3%)
Santos *et al* [67]	Adult	1986–1991	8/37 (21.6%)
Fallo *et al* [101]	Paediatric	1990–1997	11/79 (13.9%)
**Indian studies**			
Arora & Kumar [102]	Adult	1991–1997	3/12 (25.0%)
Udwadia *et al* [53]	Adult	2000–2003	6/38 (15.8%)
Sharma *et al* [54]	Adult	2000–2003	4/10 (40.0%)
Rupali *et al* [19]	Adult	1997–1998	1/7 (14.3%)
Kumarasamy *et al* [60]	Adult	1996–2001	21/36 (58.3%)
George *et al* [65]	Adult	1993–1995	1/1 (100.0%)
Usha *et al* [103]	Adult	NR	1/9 (11.1%)
Giri *et al* [84]	Combined	1986–1993	7/9 (77.8%)
Merchant *et al* [104]	Paediatric	1994–2000	2/11 (18.2%)
Madhivanan *et al* [105]	Paediatric	1996–2000	1/5 (20.0%)

Combined = both adult and paediatric populations.

### PCP Prophylaxis Appears Effective

Three adult studies [28], [37], [38] (total 445 patients) and four paediatric studies [21], [33], [34], [39] (total 524 patients) detailed prophylaxis use amongst patients with PCP in resource-poor settings. Table 8 details odds ratios and quality assessments for these studies. Data is not pooled due to differences in study design and populations. All studies indicated reduced odds for PCP amongst patients taking prophylaxis, although only three significantly so.

**Table 8:** Summary of studies detailing effects of prophylaxis on prevalence of PCP.

Study	Population	Country/Setting	Taking prophylaxis		Not taking prophylaxis		OddsRatio	95% confidence interval	Quality assessments		
			PCPcases	Total	PCPcases	Total			SelectionScore (max 4)	Comparabilityscore (max 2)	Diagnostic score (max 6)
**Anekthananon ** ***et al*** **,** **2004 ** [**37**]	HIV-infected adults, hospitalisations.	Thailand.University hospital.	6	61	35	165	0.41	0.16–1.02	2	2	NR
**Knauer ** ***et al*** **, 2005** [**38**]	HIV-infected adults, interstitial shadows on CXR.	Thailand.Tertiary hospital.	3	24	12	35	0.27	0.07–1.11	2	2	1
**Le Minor ** ***et al*** **, 2008** [**28**]	HIV-infected adults, clinical lung infection and CXR changes	Cambodia.Tertiary hospital.	3	33	81	127	0.06	0.02–0.20	4	2	6
**Madhi ** ***et al*** **, 2002** [**21**]	HIV-infected children, severe pneumonia	South Africa.Public hospital.	26	69	75	172	0.78	0.44–1.39	4	2	6
**Zar ** ***et al*** **, 2001** [**78**]	Children with pneumonia/severe pneumonia or HIV-infected and admitted to ICU	South Africa.University hospital.	1	59	15	93	0.09	0.01–0.70	2	2	6
**Chokephaibulkit ** ***et al*** **,** **1999 ** [**33**]	HIV-infected children, severe pneumonia	Thailand.University hospital.	2	9	7	17	0.41	0.06–2.58	2	2	6
**Ruffini & Madhi,** **2002 ** [**34**]	Children (<2 yrs), ‘clinical HIV’ andsevere pneumonia	South Africa.Secondary andtertiary hospital.	14	40	37	65	0.41	0.18–0.92	4	2	6

PCP = *Pneumocystis jirovecii* pneumonia; HIV = Human Immunodeficiency Virus; CXR = Chest X-ray; ICU = Intensive Care Unit.

### CD4 Count at Presentation with PCP is Generally <200×10^3^/ml, but is Higher in LMIC than in High Income Countries

Table 9 details available data on CD4 count at presentation with PCP. Most studies report a mean or median CD4 count <200×10^3^/ml in adults. Certain populations were documented to have higher CD4 counts: Malaysian intravenous drug users [40] (mean = 576×10^3^/ml) and [41] (range 310-1681×10^3^/ml), and Filipino commercial sex workers [42] (mean = 364×10^3^/ml). However, we are unable to confirm diagnosis in these studies: two report the use of ‘sputum’, for either ‘staining’ [40] or immunofluorescence [42], but not how it was obtained. Median CD4 counts at presentation with PCP in adults in LMIC were higher than in high income countries (p = 0.028, Mann-Whitney U-test, excluding [41] as a clear outlier).

**Table 9:** CD4 count at presentation with *Pneumocystis jirovecii* pneumonia in HIV-infected people.

Low and middle income countries					
Study	Country	Adult/Paediatric	Mean(×10^3/^ml or %)	Median(×10^3/^ml or %)	Range
Kay-Thwe-Han *et al* [79]	Myanmar	Adult	132.3	–	0–562
Wood *et al* [40] 1	Malaysia	Adult	576 (IVDU patients); 65 (other patients)	–	–
von Oosterhout *et al* [80], [81]	Malawi	Adult	42.5	–	1–103
Manaloto *et al* [42]	Philippines	Adult	364	–	–
Aderaye *et al* [82]	Ethiopia	Adult	59	37	–
Yoong & Cheong [41] 1	Malaysia	Adult	–	719 [calculated]	310–1681
Swasdisevi [69]	Thailand	Adult	–	15 [calculated]	10, 20 **[only recorded twice]**
Malin *et al* [29]	Zimbabwe	Adult	–	134	5–355
Udwadia *et al* [53]	India	Adult	–	96	–
Sharma *et al* [54]	India	Adult	–	38	–
Kumarasamy *et al* [60]	India	Adult	–	87	–
Nissapatorn *et al* [66]	Malaysia	Adult	–	16.5	–
Lian *et al* [22]	Malaysia	Adult	–	37	–
Kibiki *et al* [83]	Tanzania	Adult	–	26	–
Giri *et al* [84]	India	Adult+Paediatric	–	6	–
Kouakoussui *et al* [23]	Ivory Coast	Paediatric	–	–	5–15%
Ruffini & Madhi [34]	South Africa	Paediatric	22.5%	–	–
Morrow *et al* [85]	South Africa	Paediatric	13.8%	–	–
Zar et al [35], [39]	South Africa	Paediatric	16.4%	871	–
**High income countries**					
**Study**	**Country**	**Adult/Paediatric**	**Mean**	**Median**	**Range**
Kim *et al* [58]	Korea	Adult	63	–	–
Bellamy *et al* [55]	Singapore	Adult	–	16.5	–
Fang *et al* [62] 2	Taiwan	Adult	–	17.5	2–193
Hung *et al* [86] 2	Taiwan	Adult	–	32	1–193
Oh *et al* [63]	Korea	Adult	–	16	–
Moses *et al* [87]	Israel	Adult+Paediatric	–	150	10–582

1 Likely overlap between these studies.

2 Likely overlap between these studies. VDU = Intravenous drug user.

## Discussion

The most significant finding from this analysis is that the odds of a PCP diagnosis as an ADI or reason for hospitalisation in the HIV-infected population are strongly and positively correlated with *per capita* GDP. Although failure to make the diagnosis in resource-poor countries may contribute to this relationship (discussed below), we hypothesise that the relative scarcity of PCP in LMIC is primarily a result of poverty. In conditions of poverty, HIV-infected individuals are exposed to many infectious agents and non-infectious health challenges resulting in hospitalisation: these risks are compounded by weak health systems with poor preventative healthcare. In situations of high ‘pathogen competition’, relatively non-pathogenic organisms such as *Pneumocystis* will be under-represented. As countries develop economically they reduce health risks and improve the conditions underlying transmission of contagious infection: as reflected in the decreasing prevalence of tuberculosis with increasing GDP (Figure 4B). Similarly, there is a highly significant negative correlation between *per capita* GDP [15] and WHO age-standardized death rates (available at http://www.who.int/healthinfo/global_burden_disease/estimates_country/en/index.html) from meningitis or lower respiratory tract infections (Pearson correlation coefficient for meningitis -0.39, p<0.0001, n = 166 countries; Pearson correlation coefficient for respiratory infection -0.43, p<0.0001, n = 168 countries), suggesting a higher burden of severe illness with virulent pathogens in poorer countries. *Pneumocystis jirovecii*, ubiquitous in all settings, thus becomes a greater relative threat with economic advancement. The implicit conclusion is that HIV-infected people in wealthy countries are ‘protected’ from early presentation, since their CD4 count can drop into the range of risk for PCP before they develop illness.

As mentioned above, another explanation for the association between risk of PCP and GDP would be improving diagnostics with economic development. This may well be important, but it is worth noting that we found no relationship between diagnostic quality and GDP in these studies. Lower quality techniques did appear to reduce odds of diagnosis (although curiously the highest odds were seen when diagnostic technique was not recorded), but inclusion of diagnostic score in our model did not significantly alter the core findings. Effects of GDP were also similar when analysis was restricted to studies with high quality diagnosis, and in African clinical studies which predominantly diagnosed PCP with certainty (Table 5). Nevertheless, it remains possible that increasing awareness of PCP among clinicians results in more investigation or clinical diagnosis: this may explain some of the apparent increase over time or differences between geographical areas. However, it seems unlikely that this phenomenon would abolish the relationship we have demonstrated with GDP, since it would imply gross under-diagnosis of PCP in LMIC. In Western Europe before ART was introduced, PCP was responsible for two thirds of ADI, and 75% of HIV-infected patients developed PCP during their lifetime [43]: if PCP existed at a similar prevalence in LMIC it would certainly be identified. In contrast, high quality autopsy and clinical studies have failed to identify high rates of PCP in low income settings [2], [3], [26]. Notably, our model predicts approximately 6.5-fold increased odds of PCP diagnosis at the GDP levels of Western European countries in the early 1980s versus low-income countries (GDP<$1000 *per capita*), consistent with observed data.

In terms of other predictors, we observed higher odds for PCP in Central/South America compared to other areas (we are cautious about Middle Eastern results due to low numbers); higher odds in children than adults; higher odds in nationwide studies than hospitals and probably the lowest in rural areas; and an increase over time. All these factors were weaker predictors than GDP. They may reflect economic differences not reflected in national GDP figures (eg greater relative poverty in rural areas, falling ‘absolute poverty’ over time, or an effect of urbanisation). Alternatively, there may be biological explanations, most plausible for the increased risk in children. Finally, they may reflect disparities in awareness among clinicians of a condition which is difficult to diagnose.

We evaluated the challenge of clinical diagnosis by assessing features which might be suggestive of PCP in resource-poor settings. We discovered several features whose absence (cough, dyspnoea, fever) or presence (haemoptysis) pointed strongly against a diagnosis of PCP, but there was little specific to PCP itself. Although ‘typical’ chest radiographic appearances varied, the chest x-ray did provide some discriminatory power in adults. In children, all predictive features performed more poorly.

Some of this diagnostic difficulty may arise from frequent co-infection. As discussed, in situations of multiple pathogen exposure we would expect virulent organisms (highly infectious and pathogenic even in immunocompetent people) to dominate. This is borne out by high rates of *M.tuberculosis* and virulent bacterial infection. Correspondingly, true ‘opportunistic’ infection was not as highly represented in this data, and was often seen in high income countries, including two of three defined non-tuberculous mycobacteria (Israel [44]) and both protozoal infections (Israel [44] and Bahrain [45]). *Cryptococcus* was identified in 63 cases, but the majority were pulmonary infection, which does not necessarily indicate significant immunosuppression (50 cases are from a single cardio-respiratory necropsy study [46]). Although CMV was frequently detected, its significance in respiratory samples from HIV-infected patients remains uncertain [47], [48].

Virulent co-infection might be one explanation for the high case fatality from PCP in LMIC (although we do not have statistical proof for this hypothesis). Overall case fatality was 30.8% across 41 studies. This appears to be higher than in high income settings: one recent US paper reported a case fatality of 8.4% amongst 524 patients [49]. Similarly, we observed a significant negative relationship between case fatality and GDP in our data. Antimicrobial therapy against PCP seemed to be largely appropriate in these studies, but this by itself appears insufficient to prevent fatality.

This high fatality emphasises the importance of preventing PCP in these settings. Little evidence was available for ART, but more robust evidence was found for the effectiveness of prophylaxis. However, 8% of adults and 24% of children with PCP in these settings reported a history of prophylaxis receipt; use of prophylaxis also poorly predicted the absence of PCP in children (Figure 5). Practitioners should thus consider PCP even with a history of prophylaxis. Current thresholds for commencing PCP prophylaxis seem appropriate, as the majority of PCP patients in LMIC have significant cell-mediated deficiency (Table 9). However, median CD4 counts in LMIC were higher than in high income countries, and there was weak evidence that additional risk factors (intravenous drug use or commercial sex work) might predispose to PCP at higher CD4 count.

The effectiveness of ART and prophylaxis in preventing PCP potentially contradicts the negative relationship between GDP and risk of PCP, since intuitively higher income countries have greater access to these interventions. However, this is probably explained by the fact that PCP is often the presenting illness in HIV-infected individuals.

### Limitations

The search only included three databases, but identified a large number of studies and is therefore probably comprehensive; however, we may have missed studies with no recorded cases of PCP. Inclusion of countries further outside tropical latitude might have strengthened the relationship with GDP, although we note that PCP is three times commoner as an ADI in Western Europe than Eastern Europe [50], which is consistent with our data. Accurate population denominators were not available for the included studies, thus results indicate effects on the relative rather than absolute frequency of PCP diagnosis. However, areas differ widely in HIV prevalence, and the relative frequency of complications defines the priorities for policy-makers responsible for a defined population.

## Conclusions

These data should alert medical practitioners and policy-makers. PCP is likely to increase in relative frequency as an HIV complication in LMIC as they develop economically, and physicians should familiarise themselves with the presentations of this infection. Prophylaxis against PCP appears effective and should be provided according to guidelines. Case fatality from PCP is high in LMIC settings, and demands improvement.

Increasing odds of PCP with rising GDP implies that wealthier economic environments allow the CD4 count to fall further than in LMIC before HIV infection presents. This emphasises the importance in all settings of identifying HIV infection early. Those presenting with PCP usually have a CD4 count <200×10^3^/ml, and low nadir CD4 predicts poorer long term prognosis from HIV infection [51].

In summary, we have demonstrated a link between GDP and diagnosis of PCP, and thereby provide an explanation for the previously-described epidemiology in tropical and low income settings.

## Supporting Information

Checklist S1 PRISMA checklist. (DOC)

References S1 Additional references reviewed and analysed but not specifically cited in the manuscript. (DOCX)
