# Supplementary material for: Pneumocystis jirovecii Pneumonia in Tropical and Low and Middle Income Countries: A Systematic Review and Meta-Regression
Source: PLoS One. 2013 Aug 2;8(8):e69969. doi: 10.1371/journal.pone.0069969 (PMC3732248; doi:10.1371/journal.pone.0069969)
Supplement: References S1 — (DOCX) [file pone.0069969.s002.docx]

**References S1**

**These papers were reviewed for the study but are not specifically cited.**

106. Bii CC, Kose J, Taguchi H, Yepthomi T, Venkatesan C *et al*. (**2006**) Pneumocystis jirovecii and microbiological findings in children with severe pneumonia in Nairobi, Kenya. Int J Tuberc Lung Dis 10(11): 1286-91.

107. Lockman S, Hone N, Kenyon TA, Mwasekaga M, Villauthapillai M *et al*. (**2003**) Etiology of pulmonary infections in predominantly HIV-infected adults with suspected tuberculosis, Botswana. Int J Tuberc Lung Dis 7(8): 714-23.

108. Lodha R, Singhal T, Jain Y, Kabra SK, Seth P *et al*. (**2000**) Pediatric HIV infection in a tertiary care center in north India: early impressions. Indian Pediatr 37: 982-6.

109. Mahomed AG, Murray J, Klempman S, Richards G, Feldman C *et al*. (**1999**) Pneumocystis carinii pneumonia in HIV infected patients from South Africa. East Afr Med J 76(2): 80-4.

110. McLeod DT, Neill P, Gwanzura L, Latif AS, Emmanuel JC *et al*. (**1990**) *Pneumocystis carinii* pneumonia in patients with AIDS in Central Africa. Respir Med 84(3): 225-8.

111. Sun HY, Chen MY, Hsieh SM, Sheng WH, Chang SY *et al*. (**2006**) Changes in the clinical spectrum of opportunistic illnesses in persons with HIV infection in Taiwan in the era of highly active antiretroviral therapy. Jpn J Infect Dis 59(5): 311-6.

112. Michalany J, Mattos ALA, Michalany NS, Filie AC, Montezzo LC (**1987**) Acquired immune deficiency syndrome (AIDS) in Brazil. Necropsy findings. Ann Pathol 7(1): 15-24.

113. Mohar A, Romo J, Salido F, Jessurun J, Ponce de Leon S *et al*. (**1992**) The spectrum of clinical and pathological manifestations of AIDS in a consecutive series of autopsied patients in Mexico. AIDS 6(5): 467-73.

114. Murray J, Sonnenberg P, Nelson G, Bester A, Shearer S *et al*. (**2007**) Cause of death and presence of respiratory disease at autopsy in an HIV-1 seroconversion cohort of southern African gold miners. AIDS 21(Suppl 6): S97-104.

115. Nathoo KJ, Gondo M, Gwanzura L, Mhlanga BR, Mavetera T *et al*. (**2001**) Fatal Pneumocystis carinii pneumonia in HIV-seropositive infants in Harare, Zimbabwe. Trans R Soc Trop Med Hyg 95(1): 37-9.

116. Nelson AM, Perriens JH, Kapita B, Okonda L, Lusamuno N *et al*. (**1993**) A clinical and pathological comparison of the WHO and CDC case definitions for AIDS in Kinshasa, Zaire: is passive surveillance valid? AIDS 7(9): 1241-5.

117. Lanjewar DN, Duggal R (**2001**) Pulmonary pathology in patients with AIDS: an autopsy study from Mumbai. HIV Med 2(4): 266-71.

118. Worodria W, Okot-Nwang M, Yoo SD, Aisu T (**2003**) Causes of lower respiratory infection in HIV-infected Ugandan adults who are sputum AFB smear-negative. Int J Tuberc Lung Dis 7(2): 117-23.

119. Xu KF, Lu W, Li L, Shen R (**2000**) Pulmonary complications in patients with AIDS: a report from a Beijing hospital. Respirology 5(4): 419-21.

120. Yoo JH, Lee DG, Choi SM, Choi JH, Park YH *et al*. (**2004**) Infectious complications and outcomes after allogeneic hematopoietic stem cell transplantation in Korea. Bone Marrow Transplant 34(6): 497-504.

121. Tomashefski JF Jr, Butler T, Islam M. (**1989**) Histopathology and etiology of childhood pneumonia: an autopsy study of 93 patients in Bangladesh. Pathology 21(2): 71-8.

122. Shah SR, Tullu MS, Kamat JR (**2005**) Clinical profile of pediatric HIV infection from India. Arch Med Res 36(1): 24-31.

123. Kamanfu G, Mlika-Cabanne N, Girard PM, Nimubona S, Mpfizi B *et al*. (**1993**) Pulmonary complications of human immunodeficiency virus infection in Bujumbura, Burundi. Am Rev Respir Dis 147(3): 658-63.

124. Kee T, Lu YM, Vathsala A (**2004**) Spectrum of severe infections in an Asian renal transplant population. Transplant Proc 36(7): 2001-3.

125. Klotz SA, Nguyen HC, Van Pham T, Nguyen LT, Ngo DT *et al*. (**2007**) Clinical features of HIV/AIDS patients presenting to an inner city clinic in Ho Chi Minh City, Vietnam. Int J STD AIDS 18(7): 482-5.

126. Amornkul PN, Tansuphasawadikul S, Limpakarnjanarat K, Likanonsakul S, Young N *et al*. (**1999**) Clinical disease associated with HIV-1 subtype B' and E infection among 2104 patients in Thailand. AIDS 13(14): 1963-9.

127. Daga SR, Verma B, Gosavi DV (**1999**) HIV infection in children: Indian experience. Indian Pediatr 36(12): 1250-3.

128. Daley CL, Mugusi F, Chen LL, Schmidt DM, Small PM *et al*. (**1996**) Pulmonary complications of HIV infection in Dar es Salaam, Tanzania. Role of bronchoscopy and bronchoalveolar lavage. Am J Respir Crit Care Med 154(1): 105-10.

129. Dhurat R, Manglani M, Sharma R, Shah NK (**2000**) Clinical spectrum of HIV infection. Indian Pediatr 37(8): 831-6.

130. Ejzenberg B, Melles H, Melles C, Dias R, Baldacci ER *et al*. (**1996**) Aerobic bacteria, Chlamydia trachomatis, Pneumocystis carinii and Cytomegalovirus as agents of severe pneumonia in small infants. Rev Inst Med Trop Sao Paulo 38(1): 9-14.

131. Elvin KM, Lumbwe CM, Luo NP, Björkman A, Källenius G *et al*. (**1989**) Pneumocystis carinii is not a major cause of pneumonia in HIV infected patients in Lusaka, Zambia. Trans R Soc Trop Med Hyg 83(4): 553-5.

132. Eza D, Cerrillo G, Moore DA, Castro C, Ticona E *et al*. (**2006**) Postmortem findings and opportunistic infections in HIV-positive patients from a public hospital in Peru. Pathol Res Pract 202(11): 767-75.

133. Fonseca LA, Reingold AL, Casseb JR, Brigido LF, Duarte AJ (**1999**) AIDS incidence and survival in a hospital-based cohort of asymptomatic HIV seropositive patients in São Paulo, Brazil. Int J Epidemiol 28(6): 1156-60.

134. Gutierrez EB, Zanetta DM, Saldiva PH, Capelozzi VL (**2002**) Autopsy-proven determinants of death in HIV-infected patients treated for pulmonary tuberculosis in Sao Paulo, Brazil. Pathol Res Pract 198(5): 339-46.

135. Hsu RB, Fang CT, Chang SC, Chou NK, Ko WJ *et al*. (**2005**) Infectious complications after heart transplantation in Chinese recipients. Am J Transplant 5(8): 2011-6.

136. Ikeogu MO, Wolf B, Mathe S (**1997**) Pulmonary manifestations in HIV seropositivity and malnutrition in Zimbabwe. Arch Dis Child 76(2): 124-8.

137. Ansari NA, Kombe AH, Kenyon TA, Mazhani L, Binkin N *et al*. (**2003**) Pathology and causes of death in a series of human immunodeficiency virus-positive and -negative pediatric referral hospital admissions in Botswana. Pediatr Infect Dis J 22(1): 43-7.

138. Bardgett HP, Dixon M, Beeching NJ (**2006**) Increase in hospital mortality from non-communicable disease and HIV-related conditions in Bulawayo, Zimbabwe, between 1992 and 2000. Trop Doct 36(3): 129-31.

139. Bhoopat L, Thamprasert K, Chaiwun B, Attasiri C, Vithayasai P *et al*. (**1994**) Histopathologic spectrum of AIDS-associated lesions in Maharaj Nakorn Chiang Mai Hospital. Asian Pac J Allergy Immunol 12(2): 95-104.

140. Chintu C, Mudenda V, Lucas S, Nunn A, Lishimpi K *et al*. (**2002**) Lung diseases at necropsy in African children dying from respiratory illnesses: a descriptive necropsy study. Lancet 360(9338): 985-90.

141. Cury PM, Pulido CF, Furtado VM, da Palma FM (**2003**) Autopsy findings in AIDS patients from a reference hospital in Brazil: analysis of 92 cases. Pathol Res Pract 199(12): 811-4.

142. Vray M, Germani Y, Chan S, Duc NH, Sar B *et al*. (**2008**) Clinical features and etiology of pneumonia in acid-fast bacillus sputum smear-negative HIV-infected patients hospitalized in Asia and Africa. AIDS 22(11): 1323-32.

143. Jeena PM, Coovadia HM, Chrystal V (**1996**) Pneumocystis carinii and cytomegalovirus infections in severely ill, HIV-infected African infants. Ann Trop Paediatr 16(4): 361-8.

144. Aderaye G, Bruchfeld J, Olsson M, Lindquist L (**2003**) Occurrence of Pneumocystis carinii in HIV-positive patients with suspected pulmonary tuberculosis in Ethiopia. AIDS 17(3): 435-40.

145. Ansari NA, Kombe AH, Kenyon TA, Hone NM, Tappero JW *et al*. (**2002**) Pathology and causes of death in a group of 128 predominantly HIV-positive patients in Botswana, 1997-1998. Int J Tuberc Lung Dis 6(1): 55-63
